# Prognostic Survival Model Following Primary Radical Surgery for Early-Stage Cervical Cancer

**DOI:** 10.3390/cancers18071134

**Published:** 2026-04-01

**Authors:** Rattiya Phianpiset, Chayanid Detwongya, Manatsawee Manopunya, Chalong Cheewakriangkrai, Kittipat Charoenkwan

**Affiliations:** Division of Gynecologic Oncology, Department of Obstetrics and Gynecology, Faculty of Medicine, Chiang Mai University, Chiang Mai 50200, Thailand; rattiya.phi@cmu.ac.th (R.P.); chayanid.detw@cmu.ac.th (C.D.); manatsawee.m@cmu.ac.th (M.M.); chalong.c@cmu.ac.th (C.C.)

**Keywords:** prediction model, recurrence-free survival, risk assessment, uterine cervical neoplasms

## Abstract

**Background**: Radical hysterectomy with pelvic lymphadenectomy is a standard surgical procedure for early-stage cervical cancer. However, even with optimal treatment, some patients still experience disease recurrence. We aimed to develop and validate a prediction model to classify patients according to the risk of recurrence which can better assist clinicians to tailor the postoperative treatment and improve patient outcomes. **Methods**: Data of women diagnosed with early-stage cervical cancer who underwent radical hysterectomy were collected and analyzed. The primary outcome was recurrence-free survival (RFS). A prediction model based on Cox proportional hazard regression was developed by using the backward elimination procedure. Internal validation was performed by bootstrapping. The model’s discriminative ability was demonstrated by the concordance index (C-index). The model’s calibration was examined through a calibration plot. The final prognostic model was presented as a nomogram and a web-based calculator, which were further used to categorize patients into low, moderate, and high-risk groups for clinical application. **Results**: Among the 1309 patients, 115 (8.8%) experienced a recurrence. The median follow-up was 72.2 months. The 3-year and 5-year RFS rates were 93.0% (95% CI, 91.5–94.6%) and 90.7% (95% CI, 88.9–92.5%), respectively. Tumor size, histological subtype, number of positive lymph nodes, lymphovascular space invasion, and platelet-to-lymphocyte ratio were significantly associated with RFS. These factors were employed to construct a prediction model. The model exhibited a good overall fit with minimal overfitting and good calibration. The model’s discriminative performance, as measured by the C-index, was 0.73. **Conclusions**: Our proposed survival model offers a potentially valuable tool for therapeutic decision-making in patients with early-stage cervical cancer. This model demonstrates robust discriminative performance and predictive calibration. Nevertheless, external validation across diverse datasets should be conducted to assess the reproducibility and applicability of this predictive model across a broader spectrum of patients.

## Simple Summary

Radical hysterectomy is the standard treatment for early-stage cervical cancer, yet recurrence remains a challenge. The parameters currently used to classify risk groups are insufficient for providing a high-precision, individualized prognosis. We developed a comprehensive prognostic model by integrating traditional pathological factors with hematological markers. Analyzing data from 1309 patients, our model provides personalized risk prediction and categorizes patients into three distinct risk groups. Notably, the model identified high-risk features in around 10% of patients previously classified as “low risk” by traditional pathology. This multi-parametric approach provides a more precise tool for personalizing postoperative management and surveillance strategies in early-stage cervical cancer.

## 1. Introduction

Cervical cancer remains one of the most common gynecologic malignancies worldwide and continues to represent a major public health burden, particularly in low- and middle-income countries [1]. For patients diagnosed with early-stage disease (generally stages I–IIA), radical hysterectomy with pelvic lymphadenectomy remains a standard curative treatment, offering favorable long-term survival outcomes [2,3]. Nevertheless, despite apparently localized disease and optimal surgical management, approximately 10–15% of patients experience disease recurrence. Once recurrence occurs, prognosis is generally poor, and therapeutic options are limited [4]. Therefore, accurate identification of patients at increased risk of recurrence is essential to optimize postoperative management and surveillance strategies.

Risk stratification following radical surgery for early-stage cervical cancer has traditionally been based on histopathological findings obtained from surgical specimens. A landmark study conducted by the Gynecologic Oncology Group (GOG) established the Sedlis criteria, which define a population with intermediate risk of recurrence based on tumor size, depth of stromal invasion, and lymphovascular space invasion (LVSI), and demonstrated that patients categorized as intermediate risk benefit from adjuvant radiation therapy [5]. In contrast, the identification of high-risk pathological factors, including pelvic lymph node metastasis, parametrial invasion, or positive surgical margins, typically indicates the need for adjuvant concurrent chemoradiotherapy (CCRT) [6]. Although these criteria have long guided postoperative management, such categorical risk stratification may not fully capture the continuous and patient-specific nature of recurrence risk [7].

In recent years, prognostic modeling approaches have been increasingly developed to improve individualized risk prediction in cervical cancer. Multivariable prediction models integrate multiple clinicopathological variables, allowing estimation of patient-specific recurrence probabilities rather than relying solely on predefined risk categories [8]. Variables frequently incorporated into these models include tumor size, histological subtype, lymph node status, depth of stromal invasion, LVSI, and parametrial involvement [8,9]. In addition, systemic inflammatory biomarkers such as the neutrophil-to-lymphocyte ratio (NLR) and platelet-to-lymphocyte ratio (PLR) have emerged as potential prognostic indicators reflecting tumor-associated inflammatory responses [10]. Importantly, most of these parameters are routinely obtained from standard pathological reports and preoperative laboratory tests, making them readily available and cost-effective even in resource-limited healthcare settings. Several prediction models have demonstrated promising performance, with reported concordance indices ranging from 0.60 to 0.80 [8]. However, many existing models are limited by moderate predictive accuracy, lack of validation, or limited accessibility for routine clinical use.

Given these considerations, further efforts are needed to develop robust prognostic tools that integrate established pathological factors with readily available clinical and hematological markers while remaining practical for clinical application. In the present study, we developed and internally validated a multivariable prognostic model for recurrence-free survival (RFS) in a large cohort of patients with early-stage cervical cancer treated with primary radical surgery. The model incorporates both pathological features and systemic inflammatory markers and was translated into user-friendly clinical tools, including a nomogram and an interactive web-based calculator, to facilitate individualized recurrence risk prediction and support postoperative decision-making.

## 2. Materials and Methods

### 2.1. Study Design and Populations

This is a retrospective cohort analysis of women diagnosed with early-stage cervical cancer who underwent primary radical surgery at Chiang Mai University Hospital, a tertiary referral center in northern Thailand. The study population comprised women aged ≥ 20 years diagnosed with clinically stage IA1 with LVSI to IIA1 cervical carcinoma (FIGO 2018 staging) [11] with squamous cell carcinoma, adenocarcinoma, or adenosquamous carcinoma histology, who underwent laparoscopic or open modified radical hysterectomy (type B), radical hysterectomy (type C) according to the Querleu and Morrow classification [12], with pelvic lymph node dissection, between January 2003 and December 2019. Eligible patients were identified through a review of institutional medical records and surgical databases.

Patients were excluded from the analysis if they met any of these conditions: (1) receiving neoadjuvant chemotherapy or radiotherapy before definitive surgery; (2) having another synchronous primary malignancy; (3) showing pathological evidence of metastatic disease involving the cervix instead of a primary cervical cancer; or (4) lacking complete clinical records for essential clinicopathologic variables.

Postoperative adjuvant therapy decisions followed institutional protocols aligned with standard risk-based management. Patients exhibiting high-risk features such as pelvic lymph node metastasis, parametrial invasion, or positive vaginal margins received adjuvant concurrent chemoradiotherapy (CCRT). The CCRT regimen included pelvic external beam radiotherapy totaling 50.4 Gy in 28 fractions, combined with weekly intravenous cisplatin at 40 mg/m^2^ for 4 to 6 cycles [6]. Patients fulfilling the Sedlis criteria, which incorporate a defined combination of LVSI, depth of stromal invasion, and tumor size, typically received adjuvant radiotherapy alone [5].

After completion of primary and adjuvant treatment, all patients were followed through a structured surveillance schedule. Follow-up visits were conducted every 3 months during the first postoperative year, every 4 months during the second year, every 6 months from the third through the fifth year, and annually thereafter.

The Research Ethics Committee of the Faculty of Medicine, Chiang Mai University, approved this study (approval number: 423/2565, 22 November 2022).

### 2.2. Outcome Definition

The primary oncologic endpoint was recurrence-free survival (RFS), defined as the time from the date of surgery to the first documented evidence of disease recurrence. Recurrence was determined based on clinical assessment, radiologic findings, and, when available, histopathological confirmation from biopsy specimens. Patients who remained free of recurrence were censored at the time of death or at the date of their last clinical follow-up.

### 2.3. Potential Variables Selection and Data Collection

Clinicopathological data were retrospectively extracted from institutional medical records, including patient and tumor characteristics, preoperative laboratory parameters, intraoperative findings, and postoperative adjuvant treatment details. Candidate covariates were selected based on clinical relevance and established prognostic significance in the existing literature.

The variables examined for potential model inclusion included demographics (age, menopausal status), tumor-specific factors (size, histology, grade, depth of stromal invasion, and LVSI), and the extent of disease (including vaginal involvement, uterine corpus invasion, parametrial invasion, and number of pelvic or para-aortic lymph node metastasis). Furthermore, we analyzed preoperative hematological and inflammatory markers, specifically hemoglobin levels (Hb), NLR, and PLR, calculated from tests performed within 30 days prior to surgery. Adjuvant treatment details were also recorded, including the administration of radiotherapy or chemotherapy.

### 2.4. Missing Data Handling

Missing data were handled using multiple imputation to minimize bias. Multiple imputation by chained equations (MICE) was applied under the assumption that data were missing at random. Ten imputed datasets were generated using predictive mean matching for continuous variables, which preserves the original data distribution and avoids imputed values outside the plausible range. We performed five iterations per imputation to achieve convergence. A density plot was used to confirm adequate overlap between observed and imputed values.

### 2.5. Model Development and Final Variable Selection

A multivariable Cox proportional hazards regression model was developed to identify independent prognostic factors for RFS following primary radical surgery. The relationship between continuous predictors and the outcome was assessed for potential non-linearity, and, if present, was accounted for using restricted cubic splines.

The full model included all clinically relevant candidate predictors identified in the prior literature. These included histological subtypes, tumor size, depth of stromal invasion, number of positive nodes, LVSI, vaginal metastasis, vaginal margin involvement, parametrial metastasis, uterine corpus metastasis, adjuvant treatment modalities, and laboratory biomarkers including Hb, NLR, and PLR. Continuous predictors were examined for potential nonlinear association with the outcome. Variables with significant non-linear effects were modeled using a restricted cubic spline. Variable selection was performed using backward selection based on Akaike’s Information Criterion (AIC) applied to the full imputed model to obtain a parsimonious model that balanced model fit and complexity. Regression coefficients for the final Cox proportional hazards model were estimated using the pooled estimates of the imputed models.

### 2.6. Internal Validation and Model’s Performance Assessment

Internal validation was performed using bootstrap resampling (300 resamples) to assess model stability and predictive performance. The optimism-corrected concordance index (C-index), calculated from a Somers’ Dxy, was used to assess the model’s discriminative performance. Subsequently, patients were stratified into three groups according to the tertiles of predicted risk. Model’s calibration was evaluated via calibration plots comparing the mean predicted probabilities of recurrence at 3 years for each group with the corresponding observed Kaplan–Meier outcome estimates. The optimism-corrected calibration slope, the observed-to-expected (O/E) event ratio, and the calibration-in-the-large (CITL), expressed as the difference between observed and predicted event probability, were examined.

### 2.7. Model Implementation and Risk Prediction

To enable individual survival prediction, the model was reconstructed to obtain the baseline survival function while retaining the pooled coefficients and variance. Individual RFS probabilities were then calculated at 36 and 60 months. Patients were stratified into low-, intermediate-, and high-risk groups based on the tertiles of the predicted risk distribution. Observed outcomes within each group were evaluated using Kaplan–Meier estimates. Decision curve analysis was performed to evaluate the potential clinical utility of the prediction model for 3-year recurrence risk.

The final multivariable Cox regression model was translated into a risk prediction tool, a graphical nomogram and an interactive web-based risk calculator. This approach allows clinicians to estimate individualized recurrence risk by combining the weighted contribution of each predictor included in the model. The nomogram representation enables straightforward calculation of predicted survival probabilities in routine clinical practice, whereas the web-based calculator enables automated risk calculation and facilitates broader clinical implementation.

All analyses were performed in R version 4.5.1 (R Foundation for Statistical Computing, Vienna, Austria, 2025). Statistical significance was set at a two-sided *p*-value of <0.05.

## 3. Results

### 3.1. Patient Characteristics and Oncological Outcomes

A total of 1309 patients met the inclusion criteria for analysis. Recurrence occurred in 115 patients (8.8%) over a median follow-up of 72.2 months. The estimated 3-year PFS rate was 93.0% (95% CI, 91.5–94.6%), and the 5-year PFS rate was 90.7% (95% CI, 88.9–92.5%) (Figure S1). Table 1 compares baseline characteristics of the patients with and without recurrence. Demographic characteristics, such as age, parity, and menopausal status, were comparable between the two groups. There were no notable differences in operative time, blood loss, surgical complications, or the number of lymph nodes resected. Regarding tumor characteristics, patients with recurrence showed a larger mean tumor size and higher rates of adeno/adenosquamous carcinoma, LVSI, deep cervical stromal invasion, and metastasis to nearby structures such as the vagina, parametrium, uterine corpus, and pelvic lymph nodes. The median PLR was also higher in patients with recurrent disease. Additionally, these patients more often received postoperative adjuvant treatments.

### 3.2. Model Development and Final Prognostic Model

In the initial full multivariable Cox regression model, histological subtype (*p* = 0.002), tumor size (*p* = 0.001), and number of positive lymph nodes (*p* < 0.001) were significantly associated with RFS.

Variable reduction was subsequently performed using backward elimination based on the AIC. Five predictors were retained in the final model: histological subtype, tumor size, number of positive lymph nodes, LVSI, and PLR. The final multivariable Cox proportional hazards model contained 7 degrees of freedom and remained statistically significant. The model demonstrated acceptable discriminative ability, with a Somers’ Dxy of 0.479, corresponding to a C-index of approximately 0.74. The regression coefficients are presented in Table 2.

### 3.3. Model Performance During Internal Validation

**Figure 1:**
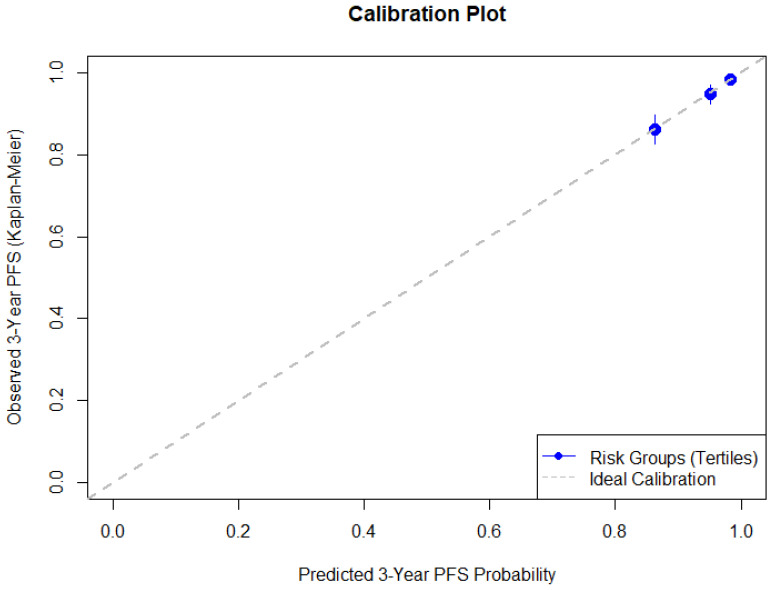
Calibration plot comparing predicted and observed 3-year recurrence-free survival.

After optimism correction, the model demonstrated good discriminative ability with a C-index of 0.73. Calibration of the prognostic model evaluated at 3 years showed good agreement between predicted and observed recurrence risk (Figure 1). The optimism-corrected calibration slope obtained from bootstrap validation was 0.847 with CITL of 0.002. The mean predicted 3-year recurrence risk was 6.8%, which was comparable to the Kaplan–Meier estimated observed recurrence risk of 7.0%. The O/E event ratio at 3 years was 1.027.

**Figure 2:**
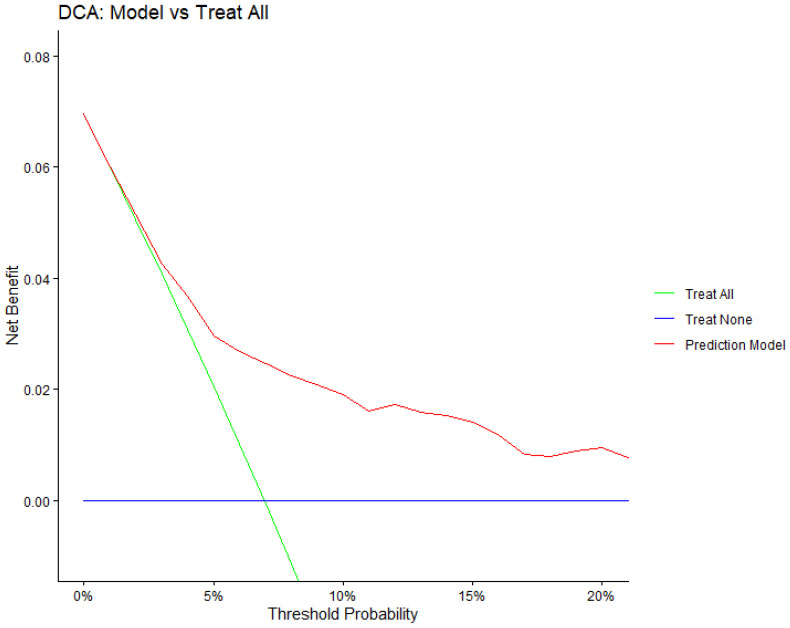
Decision curve analysis of the prognostic model for predicting 3-year recurrence risk.

From decision curve analysis, across a range of clinically plausible threshold probabilities, this model yielded a higher net benefit than both the treat-all and treat-none strategies (Figure 2), indicating that using the model to guide decision-making could reduce unnecessary interventions while maintaining detection of patients at risk of recurrence.

**Figure 3:**
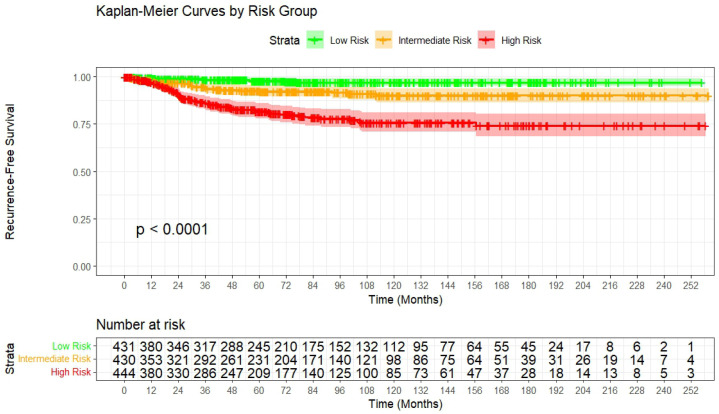
Kaplan–Meier curves of recurrence-free survival stratified by model-risk groups.

Risk stratification was performed by categorizing patients into tertiles based on the model-predicted recurrence risk. Kaplan–Meier curves demonstrated clear separation in RFS among the three risk groups (log-rank test, *p* < 0.001) (Figure 3). At 3 years, the estimated recurrence risk was 1.6% (95% CI 0.3–2.9) in the low-risk group, 5.3% (95% CI 2.9–7.6) in the intermediate-risk group, and 13.6% (95% CI 10.0–17.0) in the high-risk group. At 5 years, the corresponding recurrence risks were 2.0% (95% CI 0.5–3.5), 7.3% (95% CI 4.5–10.0), and 18.2% (95% CI 14.1–22.2), respectively.

### 3.4. Model Presentation

A nomogram was developed from the final multivariable Cox model to estimate individual probabilities of 3- and 5-year RFS (Figure S2). The nomogram incorporated histological subtype, tumor size, LVSI, number of positive lymph nodes, and PLR.

To facilitate clinical application, an interactive web-based calculator was developed (https://kcharoenkwan.shinyapps.io/Cervical_App/). Users input available clinicopathologic variables, including tumor size, histological subtype, LVSI status, number of positive lymph nodes, and PLR. The application then provides individualized estimates of 3-year recurrence risk and predicted RFS up to 5 years, along with classification into low-, intermediate-, or high-risk groups based on predefined cutoffs. The calculator also displays the projected survival curve over time to aid clinical interpretation. An example of the user interface is shown in Figure S3.

## 4. Discussion

In this retrospective cohort of 1309 women with early-stage cervical cancer treated with primary radical surgery, we developed and internally validated a multivariable prognostic model to estimate RFS. Five clinically available predictors, including tumor size, histological subtype, number of positive lymph nodes, LVSI, and PLR, were retained in the final model. The model demonstrated good discrimination after optimism correction, as indicated by a C-index of 0.73, with satisfactory calibration at 3 years, and showed potential clinical utility in decision curve analysis. Importantly, the model was implemented in practical formats (nomogram and web-based calculator), enabling individualized recurrence risk prediction and risk-group stratification, which may support postoperative decision-making and tailored surveillance.

The predictors included in the final model align closely with those commonly found in the broader cervical cancer prognostic literature. A systematic review of 77 cervical cancer prognostic models, including 29 focused on early-stage disease, identified lymph node status, FIGO stage, histologic type, and tumor size as the most frequently used predictors. Specifically, for early-stage cervical cancer, variables such as lymph node status, LVSI, depth of invasion, FIGO stage, histology, tumor size, and parametrial invasion were among the most commonly used [8]. This suggests that our model depends on predictors that are well-established links to prognosis and are routinely accessible in various clinical settings.

Tumor size remains one of the most important prognostic factors in early-stage cervical cancer and was included in our final model. A well-known postoperative risk stratification system (Sedlis criteria) combines tumor size with LVSI and depth of stromal invasion and has been shown to reduce recurrence with adjuvant pelvic radiotherapy in selected patients after radical hysterectomy [5]. In a retrospective study of 1178 patients with invasive cervical carcinoma treated with radiotherapy alone, tumor size also showed a strong relationship with local failure and disease control. For stage IB disease, the 10-year pelvic failure rate increased from 6% for tumors < 3 cm, to 15% for tumors 3–5 cm, and 30% for tumors > 5 cm (*p* = 0.0018) [13]. Consistent with this concept, data from our institution highlighted tumor size as the strongest independent prognostic factor associated with overall and recurrence-free survival in early-stage patients undergoing radical hysterectomy [14]. However, recent evidence proposed that using certain size cutoffs may not perform consistently across treatment contexts. In a cohort of 312 patients who underwent radical hysterectomy followed by adjuvant radiotherapy, the 2018 FIGO size categories (<2, 2–4, ≥4 cm) were not independently associated with disease-free survival (DFS) and overall survival (OS) [15]. Their finding supported that categorical thresholds may be less informative and that the use of continuous risk estimation in multivariable models may provide more useful risk estimation.

Nodal status is a strong predictor of recurrence [16,17]. During our initial model development, we first evaluated lymph node status as simply positive or negative. However, incorporating the number of positive lymph nodes provided better prognostic discrimination [18]. Consistent with prior evidence showing that risk increases progressively with greater nodal burden rather than shifting suddenly at a single threshold. In a large SEER-based analysis of 2222 node-positive early-stage patients, having >2 positive nodes was associated with significantly worse outcomes compared with 1–2 nodes (OS HR 1.57, 95% CI 1.35–1.83; *p* < 0.001), supporting the prognostic value of quantitative nodal burden [19]. Similarly, Olthof et al. identified a high-risk threshold of ≥4 positive nodes, with substantially worse survival (excess HR 2.4, 95% CI 1.6–3.5) compared with patients with fewer involved nodes [20]. Park et al. reported that node-positive patients remain heterogeneous even after accounting for nodal status and treatment, and proposed risk stratification approaches for node-positive disease using histology, tumor size, and parametrial involvement. However, the independent contribution of the number of metastatic nodes was not retained across multivariable models in their cohorts [21]. Overall, previous evidence indicates that using nodal burden rather than binary nodal status more accurately captures patient heterogeneity and enhances prediction, consistent with our findings.

Our finding that histological subtype significantly contributes to recurrence risk is supported by previous data showing that adenocarcinoma often has poorer outcomes than squamous cell carcinoma. In a recent propensity score–matched analysis of 714 surgically treated patients, adenocarcinoma had significantly lower 5-year survival than matched squamous cell carcinoma (5-year OS 82.1% vs. 95.2% and 5-year DFS 79.2% vs. 92.8%, *p* < 0.05). In contrast, adenosquamous carcinoma showed survival comparable to matched squamous cell carcinoma in that study, suggesting heterogeneity within “non-squamous” histology [22]. In addition, a recent Korean Gynecologic Oncology Group study (KGOG 1028), evaluating the Japanese Gynecologic Oncology Group (JGOG) prognostic model for early-stage intermediate-risk cervical cancer, highlighted the prognostic value of histology-incorporated risk stratification, supporting histology as a clinically relevant factor even within the intermediate-risk setting and for guiding postoperative management [23]. This biologic and prognostic divergence has also motivated recent efforts to build histology-specific prognostic models for early-stage cervical cancer, emphasizing that adenocarcinoma and squamous cell carcinoma may benefit from different risk modeling and potentially different adjuvant approaches rather than uniform management [24].

LVSI is a well-established adverse pathological feature in early-stage cervical cancer and provides prognostic information by reflecting early lymphatic/vascular dissemination potential. Biologically, molecular profiling suggests that LVSI is linked to pro-metastatic pathways and an altered tumor immune environment, supporting its association with metastasis and shorter RFS [25]. Notably, LVSI is also one of the three core components of the Sedlis criteria used to define intermediate-risk patients after radical hysterectomy, underscoring its long-standing clinical role in guiding adjuvant radiotherapy decisions in early-stage disease [5].

Systematic reviews and meta-analyses support the prognostic relevance of PLR in cervical cancer, although effect sizes vary across populations and treatment settings. An updated meta-analysis published in 2025 (30 cohort studies; 8597 patients) reported that elevated PLR was associated with significantly worse survival outcomes, both OS (HR 1.77, 95% CI 1.43–2.19, *p* < 0.001) and progression-free survival (PFS HR 1.69, 95% CI 1.26–2.27, *p* < 0.001). Subgroup analyses indicated a stronger prognostic signal in patients who received surgery combined with radiotherapy and in Asian cohorts, and suggested PLR thresholds >150 as potentially more prognostic [26]. Given its availability and low cost, PLR is particularly attractive for clinical implementation because it is routinely measured preoperatively as part of a standard complete blood count and can be readily applied even in resource-limited settings where advanced molecular profiling is not widely available.

Several previously reported prediction models for early-stage cervical cancer were limited by small effective sample sizes, incomplete reporting of the full model equation, and inconsistent evaluation of key performance measures. Across all cervical cancer prognostic models, only 68% reported internal validation and 58% assessed calibration, raising concerns about overfitting and transportability. In this context, our study addresses several recurring limitations noted in the systematic review by using a comparatively large cohort (1309 patients; 115 recurrences), handling missing data with multiple imputation, applying bootstrap internal validation, and reporting both discrimination (C-index) and calibration alongside clinical utility assessment. With respect to discrimination, the systematic review reported a wide range of performance across published models, with discrimination estimates ranging from 0.565 to 0.959 [8]. Our optimism-corrected C-index of 0.73 indicates good discriminative ability and is comparable to many previously reported early-stage prediction models while relying on a small set of routinely available predictors, enabling broad and low-cost implementation. Moreover, presenting the model as an interactive web-based calculator improves usability and may help overcome the common limitation of prior models regarding routine clinical implementation.

The European Society of Gynaecological Oncology (ESGO) cervical cancer recurrence risk calculator is based on the Annual Recurrence Risk Model (ARRM), created by the international Surveillance in Cervical Cancer (SCCAN) consortium. It was developed using data from 4343 surgically treated early-stage patients (2007–2016) across 20 tertiary centers on four continents. The ARRM used a Cox model and included five predictors: maximal pathologic tumor diameter, histology type, grade, number of positive pelvic lymph nodes, and LVSI, then translated the score into annual recurrence probabilities to support risk-adapted surveillance, with good discrimination (C-statistic 0.73) and clear clinical separation across increasing risk groups (5-year DFS 97.5%, 94.7%, 85.2%, and 63.3%, with 2-year DFS 15.4% in the highest-risk group) [27]. Our model showed comparable discrimination after optimism correction (C-index 0.73) and is conceptually aligned in using largely overlapping, routinely available pathological predictors (tumor size, histology, LVSI, and nodal burden), while additionally incorporating PLR as a low-cost inflammatory biomarker that may provide incremental value, particularly in resource-limited settings. Head-to-head external validation in the same population would help clarify comparative performance and the added value of PLR.

The clinical value of a prognostic model is its ability to support risk stratification. In our cohort, model-based tertiles produced three clearly separated risk groups presented by Kaplan–Meier curves (log-rank *p* < 0.0001), indicating that predicted risk corresponded to clinically observable differences in RFS over time. This separation was also evident at a clinically relevant time point. At 3 years, the estimated recurrence risks were 1.6% in the low-risk group, 5.3% in the intermediate-risk group, and 13.6% in the high-risk group. At 5 years, the corresponding risks were 2.0%, 7.3%, and 18.2%, respectively. These gradients indicate that the model can differentiate between patients who might need more intensive follow-up and those for whom de-escalated surveillance could be appropriate.

Importantly, model-derived risk groups may provide additional stratification beyond traditional pathology-based categories defined by Sedlis (intermediate-risk) and Peters (high-risk) criteria [5,6]. Comparing traditional pathological risk with model-predicted risk demonstrated some discrepancies (Table 3). Among patients initially classified as low risk based on pathology, the model reclassified 32 individuals into a high predicted-risk group. This reveals an “occult high-risk” subset that would normally be monitored with standard surveillance, even though recurrences can still occur in this low-risk group. The result indicates that the model could help identify low-risk patients who might need closer monitoring, earlier imaging, or lower thresholds for symptom-driven assessments. Whether some of these patients could also benefit from more aggressive adjuvant therapy is a critical question for future prospective studies.

Similarly, within the pathological moderate-risk group, the model identified 14 patients with low predicted risk, suggesting that some patients may be candidates for less intensive follow-up and potentially less aggressive adjuvant strategies. Nevertheless, any shift toward treatment de-escalation requires further confirmatory evidence from prospective trials. Conversely, the model identified 129 moderate-risk patients with high predicted risk, representing a subgroup that may benefit from more intensive surveillance or consideration of additional risk-adapted management strategies in future studies. This is particularly relevant given emerging evidence questioning the survival benefit of routine adjuvant radiotherapy in all intermediate-risk patients.

The optimal management of intermediate-risk disease, as defined by the traditional Sedlis criteria, remains debated, and several contemporary studies have suggested that the oncological benefit of routine adjuvant radiotherapy after radical surgery may be limited to selected patients [28]. These observations have prompted increasing interest in refining the definition of the intermediate-risk group and identifying subsets of patients who may safely omit adjuvant therapy. In this context, the ongoing international CERVANTES randomized trial is evaluating whether radical surgery alone may be non-inferior to surgery followed by adjuvant (chemo)radiotherapy in patients with intermediate-risk early-stage cervical cancer [29]. The results of this trial are expected to provide important evidence regarding whether current Sedlis-based criteria should be modified or replaced by more individualized risk stratification approaches. Consistent with this evolving perspective, a recent propensity score-adjusted study, adjuvant pelvic radiation reduced pelvic recurrence but did not demonstrate a significant improvement in overall recurrence or overall survival among patients with intermediate-risk factors [30]. More precise risk stratification within the intermediate-risk category may help identify those most likely to benefit from increased surveillance and possibly adjuvant therapy, while avoiding unnecessary interventions for lower-risk patients [31].

Finally, even within the pathological high-risk group, predicted risk remained heterogeneous, suggesting that nodal burden, tumor biology, and systemic markers may further refine prognosis beyond the presence of high-risk features alone. Taken together, these findings support that model-based stratification may complement established pathological criteria by identifying patients whose observed recurrence may not align with conventional risk groups, thereby informing more personalized follow-up intensity and generating hypotheses for future trials of risk-adapted adjuvant therapy.

A major strength of this study is the use of a large cohort with a long follow-up period, enabling robust estimation of recurrence risk after primary radical surgery. In addition, patients were managed using uniform treatment protocols aligned with established standards, which helps reduce variability in postoperative management and supports consistency in outcome assessment. Methodologically, missing data were handled using multiple imputation, and model performance was evaluated comprehensively through internal validation, reporting both discrimination and calibration along with clinical utility assessment. The final model relies on a small number of routinely available variables derived from standard pathological reports and preoperative laboratory tests, supporting feasibility and reproducibility across diverse clinical settings. Finally, implementing the model as an interactive web-based calculator enhances usability and may facilitate its translation into routine clinical practice.

Certain limitations should be acknowledged. First, the retrospective single-center design introduces the potential for selection bias and unmeasured confounding. Second, the long inclusion period may encompass temporal changes in surgical techniques, pathological reporting standards, and adjuvant therapy protocols, which could affect both predictor distributions and baseline recurrence hazards. Another limitation is the inclusion of patients with stage IB3 and IIA2 disease. According to the FIGO guidelines for cervical cancer [2], CCRT is generally the preferred treatment for stage IB3–IIA2 cervical cancer. However, primary surgical management for selected IB3 or IIA2 cases is still practiced in our center and in some other Asian countries. While our inclusion reflects current clinical practice in our setting, the model’s applicability to strictly defined early-stage disease (≤IB2) warrants further validation. Finally, although internal validation demonstrated good calibration and discrimination, external validation is necessary before broader clinical adoption. Model performance may differ across cohorts with varying case mixes, histological distributions, surgical approaches, and adjuvant treatment policies.

Future research should focus on external validation in independent datasets, including multicenter cohorts. If validated, the model could be assessed prospectively to determine whether risk-adapted surveillance improves clinically meaningful outcomes, such as earlier detection of treatable recurrence or optimized resource utilization. Additional extensions may include the incorporation of emerging biomarkers, imaging-derived metrics, or molecular features to improve model performance. However, maintaining affordability should remain a priority, particularly in settings where cervical cancer burden is prevalent. Overall, this model represents a step toward individualized postoperative risk prediction after radical surgery for early-stage cervical cancer and provides a practical foundation for future validation and implementation studies.

## 5. Conclusions

In conclusion, we developed and internally validated a multivariable prognostic model for RFS in a large cohort of women with early-stage cervical cancer treated with primary radical surgery. The final model, incorporating tumor size, histological subtype, number of positive lymph nodes, LVSI, and PLR, demonstrated acceptable discriminative ability (optimism-corrected C-index 0.73) with good calibration at 3 years. By providing individualized recurrence risk estimates and stratifying patients into distinct risk groups, the model may complement conventional pathology-based risk classification and support more personalized postoperative surveillance strategies. Implementing the model as both a nomogram and an interactive web-based calculator enhances clinical usability. External validation in independent and diverse cohorts is warranted prior to broader adoption. Prospective studies are needed to determine whether model-guided, risk-adapted management improves patient outcomes.

## Figures and Tables

**Table 1:** Clinical characteristics of the study population.

	Disease Free (n = 1194)	Recurrence (n = 115)	*p*-Value
Age	47.31 ± 9.03	46.32 ± 9.30	0.26
Parity	2.00 [1.00, 2.00]	2.00 [1.00, 2.00]	0.07
Menopause	401 (33.58)	36 (31.30)	0.74
Prior conization	497 (41.62)	20 (17.39)	<0.001 *
Hemoglobin	12.70 [11.70, 13.50]	12.30 [11.03, 13.20]	0.04 *
NLR	1.86 [1.42, 2.58]	2.12 [1.60, 2.64]	0.06
PLR	128.32 [100.40, 161.68]	141.67 [116.67, 180.99]	0.003 *
Operative time	214.50 [188.00, 246.00]	223.00 [197.00, 243.00]	0.23
Operative blood loss	400.00 [200.00, 700.00]	400.00 [250.00, 600.00]	0.70
Operative complication	121 (10.13)	6 (5.22)	0.13
Number of lymph nodes obtained	25.90 ± 9.75	25.12 ± 9.57	0.41
Final histology			<0.001 *
- Squamous cell carcinoma	830 (69.51)	61 (53.04)	
- Adenocarcinoma	283 (23.70)	37 (32.17)	
- Adenosquamous carcinoma	81 (6.78)	17 (14.78)	
Tumor size in cm	1.89 ± 1.77	3.35 ± 1.70	<0.001 *
Tumor grade			0.13
- 1	208 (17.42)	26 (22.61)	
- 2	496 (41.54)	54 (46.96)	
- 3	130 (10.89)	24 (20.87)	
LVSI	545 (45.64)	86 (74.78)	<0.001 *
Depth of invasion			<0.001 *
- Inner 1/3	139 (11.64)	4 (3.48)	
- Middle 1/3	183 (15.33)	14 (12.17)	
- Outer 1/3	547 (45.81)	92 (80.00)	
Vaginal margin status			0.25
- Negative	1079 (90.37)	98 (85.22)	
- Positive intraepithelial lesion	67 (5.61)	9 (7.83)	
- Positive cancer	44 (3.69)	7 (6.09)	
Vaginal metastasis			<0.001 *
- Negative	955 (79.98)	77 (66.96)	
- Positive cancer	143 (11.98)	30 (26.09)	
- Positive HSIL	92 (7.71)	6 (5.22)	
Parametrium metastasis	212 (17.76)	43 (37.39)	<0.001 *
Uterine metastasis			<0.001 *
- Negative	1057 (88.53)	86 (74.78)	
- Positive cancer	107 (8.96)	23 (20.00)	
- Positive HSIL	25 (2.09)	4 (3.48)	
Number of positive lymph nodes	0.00 [0.00, 0.00]	0.00 [0.00, 2.00]	<0.001 *
Postoperative chemotherapy	346 (28.98)	61 (53.04)	<0.001 *
Postoperative radiation			<0.001 *
- No	597 (50.00)	32 (27.83)	
- Brachytherapy alone	37 (3.10)	1 (0.87)	
- Whole pelvic radiation +/− brachytherapy	307 (25.71)	50 (43.48)	
FIGO 2018 staging			<0.001 *
- IA1 with positive LVSI	101 (8.46)	2 (1.74)	
- IA2	87 (7.29)	2 (1.74)	
- IB1	385 (32.24)	15 (13.04)	
- IB2	205 (17.17)	21 (18.26)	
- IB3	47 (3.94)	10 (8.70)	
- IIA1	58 (4.86)	11 (9.57)	
- IIA2	9 (0.75)	1 (0.87)	
- IIB	113 (9.46)	15 (13.04)	
- IIIC1(p)	187 (15.66)	38 (33.04)	

* Statistically significant *p* < 0.05. Continuous variables are expressed as mean ± SD for normally distributed data and median [IQR] for non-normally distributed data. Categorical variables are presented as frequency (n) and percentage (%). SD: standard deviation, IQR: interquartile range, NLR: neutrophil-to-lymphocyte ratio, PLR: platelet-to-lymphocyte ratio, LVSI: lymphovascular space invasion, HSIL: high-grade squamous intraepithelial lesion, FIGO: International Federation of Gynecology and Obstetrics.

**Table 2:** Final variables in the multivariable Cox proportional hazards model for recurrence-free survival.

Variable	β Coefficient	Hazard Ratio (HR)	95% CI	*p*-Value
Tumor size (cm)	0.547	1.73	1.20–2.51	0.004 *
Histology				
- Squamous cell carcinoma	Ref			
- Adenocarcinoma	0.703	2.02	1.32–3.09	0.001 *
- Adenosquamous carcinoma	0.713	2.04	1.19–3.51	0.01 *
Lymphovascular space invasion	0.482	1.62	1.01–2.59	0.04 *
Number of positive lymph nodes	0.089	1.09	1.04–1.14	<0.001 *
Platelet-to-lymphocyte ratio	0.0005	1.001	0.999–1.002	0.38

* Statistically significant *p* < 0.05. CI: confidence interval.

**Table 3:** Agreement between the pathological risk group and the model-predicted risk stratification.

	Model Predicting Risk		
Pathological Risk	Low	Moderate	High
Low	115	174	32
Intermediate	14	92	129
High	34	131	269

## Data Availability

The data presented in this study are available upon request from the corresponding author (the data are not publicly available due to privacy or ethical restrictions).

## Supplementary Materials

The following supporting information can be downloaded at: https://www.mdpi.com/article/10.3390/cancers18071134/s1, Figure S1: Kaplan–Meier curve of recurrence-free survival in the overall cohort; Figure S2: Nomogram for predicting 3-year and 5-year recurrence-free survival after primary radical surgery for early-stage cervical cancer; Figure S3: User interface of the web-based calculator for individualized prediction of 3-year recurrence risk after primary radical surgery for early-stage cervical cancer.
