# Supplementary figures and images for: Prognostic Survival Model Following Primary Radical Surgery for Early-Stage Cervical Cancer
Source: Cancers (Basel). 2026 Apr 1;18(7):1134. doi: 10.3390/cancers18071134 (PMC13072203; doi:10.3390/cancers18071134)

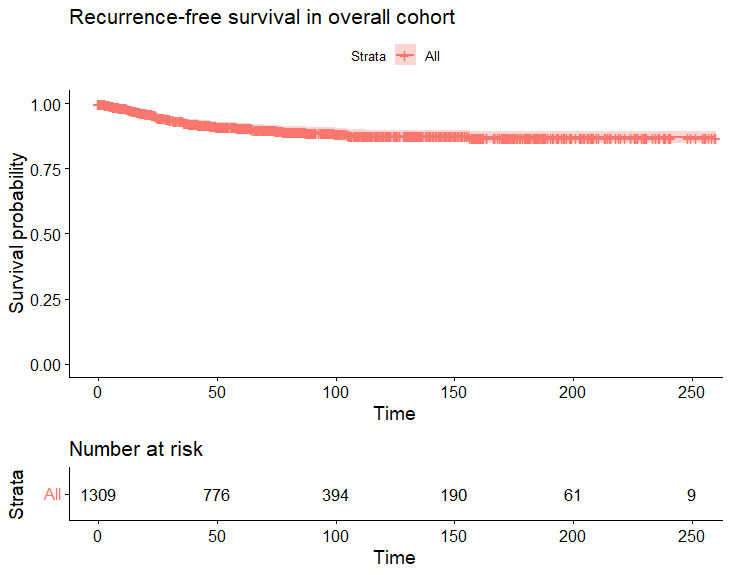

Supplement: Supplementary file 1 [file cancers-18-01134-s001.zip › Figure S1 KM curve.png]

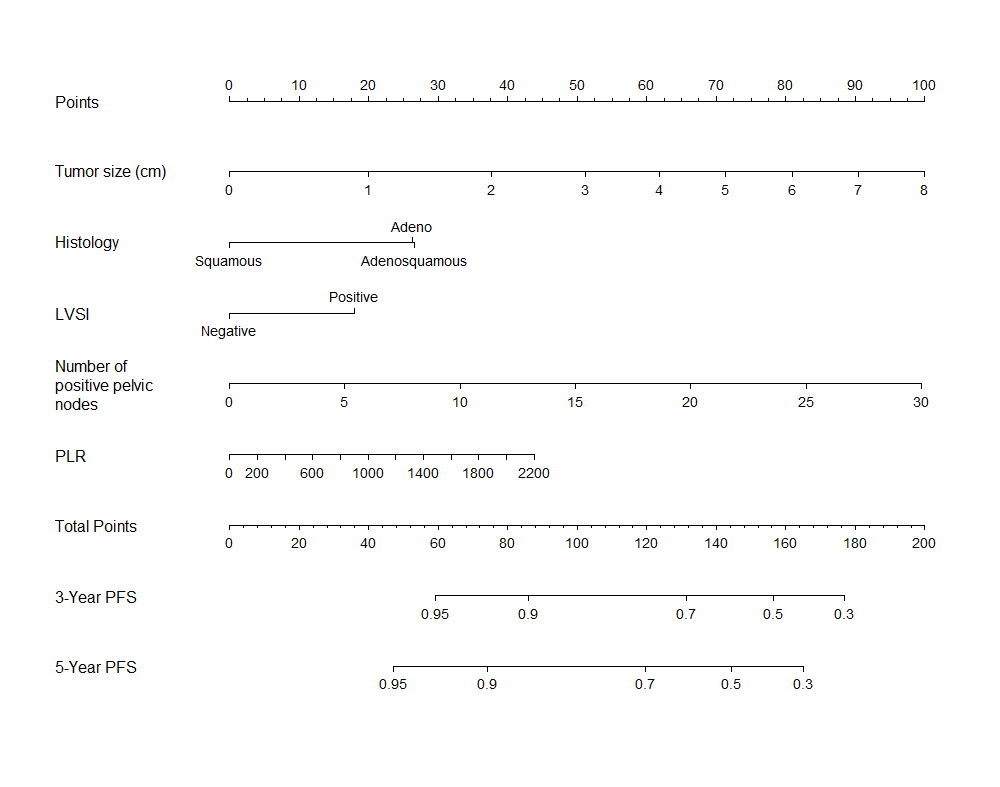

Supplement: Supplementary file 1 [file cancers-18-01134-s001.zip › Figure S2 Nomogram .png]

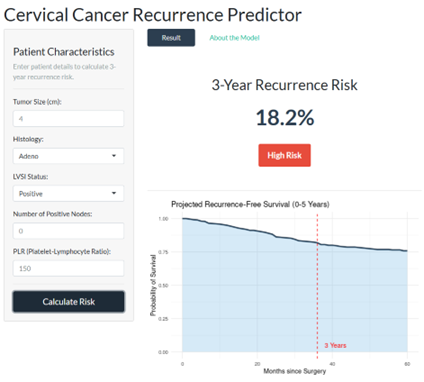

Supplement: Supplementary file 1 [file cancers-18-01134-s001.zip › Figure S3 Web calculator.png]
